# Exploring the Adhesion Potential of *Bifidobacterium bifidum* Strains Isolated from Infant Feces

**DOI:** 10.3390/foods15152659

**Published:** 2026-07-29

**Authors:** Yifan Wu, Lihong Xiao, Shirong Ding, Mingxue He, Hong Shao, Tiejun Zhao, Lili Zhang, Shuang Zhang

**Affiliations:** 1 Key Laboratory of Dairy Science, Ministry of Education, College of Food Science, Northeast Agricultural University, Harbin 150030, China; wuyf_hit@hotmail.com (Y.W.); 15248328273@163.com (L.X.); dsrkybdx@163.com (S.D.); hmx18846760418@163.com (M.H.); 2 Heilongjiang Green Food Science Research Institute, Harbin 150030, China; shaohong747@163.com; 3 Agro-Food Science, Niigata Agro-Food University, Hiranedai 2416, Tainai 959-2702, Niigata, Japan; tiejun-zhao@nafu.ac.jp

**Keywords:** *Bifidobacterium*, pathogen, Caco-2 cells, mucin, adhesion

## Abstract

Bifidobacteria capable of metabolizing human milk oligosaccharides (HMOs) regulate microbial balance and enhance immune defenses, yet their adhesive properties and pathogen-inhibitory capacity remain understudied. We examined auto-aggregation, surface hydrophobicity, adhesion, and pathogen antagonism in 12 HMO-metabolizing bifidobacterial strains isolated from infant feces, using *Bifidobacterium longum* subsp. *infantis* ATCC 15697 (BI_15697) and M63 (BI_M63) as reference strains. *Bifidobacterium bifidum* BB_Y10 and BB_Y22 exhibited the highest auto-aggregation, hydrophobicity, and adhesion. Adhesion assays using Caco-2 cells and mucin, combined with scanning electron microscopy (SEM) for qualitative morphological observation, revealed distinct surface characteristics among the strains. These morphological differences may be associated with, but do not directly establish, the observed variability in adhesion capacity. Surface-associated molecules, including surface layer proteins (SLPs), extracellular polysaccharides (EPS), and lipoteichoic acid (LTA), differentially contributed to adhesion across the strains. BB_Y10 and BB_Y22 demonstrated strong adhesive capacity and enhanced pathogen antagonism in competitive, exclusion, and displacement assays in vitro, suggesting their potential to inhibit pathogen colonization in future in vivo studies and identifying them as promising probiotic candidates for further development.

## 1. Introduction

*Bifidobacterium* is a predominant beneficial genus in the infant gut microbiota, where its colonization is a prerequisite for exerting probiotic effects, including pathogen inhibition, immune modulation, and metabolic support [1,2,3]. Successful establishment in the gut relies on robust epithelial attachment, a strain-specific property determining the residence time and bioactivity of probiotics [4,5].

Adherence to intestinal epithelial cells is a key characteristic of probiotics and is essential for their beneficial effects. Bifidobacteria utilize surface molecules to interact with the gastrointestinal (GI) lining, facilitating mucosal attachment and long-term gut residence [6]. These surface structures, referred to as adhesins or ligands, include extracellular polysaccharides (EPS), pili and various surface proteins [7].

Human milk oligosaccharides (HMOs) are the third-most abundant solid component in breast milk and serve as selective prebiotics that enrich bifidobacterial populations in the infant gut [8,9]. *Bifidobacterium longum* subsp. *infantis* and *B. bifidum* are among the primary HMO utilizers, which possess specialized glycosyl hydrolases and transporters for HMO degradation [10,11,12]. Previous studies have shown that HMO-metabolizing bifidobacteria exhibit enhanced colonization capacity, competitive exclusion of pathogens, and immunomodulatory effects in infants [13,14]. However, HMO utilization is increasingly recognized as a desirable trait for probiotic selection, the strain-specific adhesive properties of HMO-metabolizing isolates and their surface molecular determinants remain to be systematically characterized.

Emerging evidence indicates that HMO utilization can enhance bifidobacterial adhesion to intestinal epithelia. For instance, 2′-fucosyllactose (2′-FL) was shown to promote *B. bifidum* DNG6 adhesion to Caco-2 cells [15], and HMO-cultured bifidobacteria isolated from infants have been reported to enhance intestinal epithelial adherence [16]. However, these studies have primarily focused on single or limited strains under HMO-supplemented conditions, without systematic comparison of strain-specific adhesive phenotypes across a diverse set of HMO-utilizing isolates. Moreover, the surface molecular determinants (SLPs, LTA, EPS) underlying differential adhesion among HMO-metabolizing strains remain uncharacterized, and whether enhanced adhesion translates into effective pathogen antagonism through competitive, exclusion, and displacement mechanisms has not been investigated. Infant feces represent an ecological reservoir of bifidobacterial strains adapted to the HMO-rich infant gut, where co-evolution with host-derived glycans may have driven the selection of optimized adhesion systems [17]. Therefore, a systematic evaluation of the adhesive properties, surface molecular mechanisms, and pathogen-inhibitory capacity of multiple HMO-metabolizing isolates from this niche is warranted to advance the rational strain selection for probiotic applications.

In our previous study, we isolated 12 bifidobacterial strains from fecal samples of healthy, breastfed, term infants and confirmed their HMO utilization capacity and probiotic potential [18,19]. Building upon this, the present study systematically evaluated the adhesive properties of these HMO-metabolizing strains, including auto-aggregation, surface hydrophobicity, adhesion to Caco-2 cells and mucin, and antagonistic effects against enteric pathogens. By identifying surface-associated molecules (SLPs, LTA, EPS) that differentially influence adhesion, we aimed to elucidate the strain-specific mechanisms underlying mucosal colonization and pathogen inhibition, thereby advancing the rational selection of bifidobacteria optimized for intestinal adherence.

## 2. Materials and Methods

### 2.1. Bacterial Strains and Growth Conditions

*Bifidobacterium longum* subsp. *infantis* ATCC 15697 (BI_15697) was purchased from the China Center of Industrial Culture Collection (CCICC, Beijing, China), while *Bifidobacterium longum* subsp. *infantis* M63 was isolated from commercial ProTectis drops (Bio Gaia, Stockholm, Sweden).

The 12 bifidobacterial strains (BB_Y10, BB_Y22, BB_Y30, BP_Y43, BB_S7, BB_S15, BB_S40, BB_H4, BB_H5, BB_H22, BP_YA, and BL_S34) were originally isolated from fecal samples of healthy, full-term, breastfed infants in northeastern China. These strains were previously screened and identified by our research group based on their ability to utilize human milk oligosaccharides (HMOs) and galacto-oligosaccharides (GOS). Specifically, seven strains (BB_Y10, BB_Y30, BB_S40, BB_H4, BB_H5, BB_H22, and BI_Y46) were screened using 2′-fucosyllactose (2′-FL) as the sole carbon source, and their probiotic properties—including extracellular polysaccharide production, surface protein content, auto-aggregation ability, bile salt tolerance, and bacteriostatic activity—were characterized [18]. Additionally, seven strains with high GOS utilization capacity, including *B. pseudocatenulatum* (BP_Y43, BP_YA), *B. bifidum* (BB_Y22, BB_S7), and *B. longum* (BL_S34), among others, were screened, and their probiotic characteristics were evaluated [19]. In the present study, these HMO- and GOS-utilizing strains were selected to investigate their adhesive properties and antagonistic effects against enteric pathogens. All the bacteria used in this study was listed in Table 1.

**Table 1:** Bacteria used in this study.

Bacteria	Abbreviation	Origin
*Bifidobacterium bifidum* Y10	BB_Y10	Infant feces
*Bifidobacterium bifidum* Y22	BB_Y22	Infant feces
*Bifidobacterium bifidum* Y30	BB_Y30	Infant feces
*Bifidobacterium pseudocatenulatum* Y43	BP_Y43	Infant feces
*Bifidobacterium bifidum* S7	BB_S7	Infant feces
*Bifidobacterium bifidum* S15	BB_S15	Infant feces
*Bifidobacterium bifidum* S40	BB_S40	Infant feces
*Bifidobacterium bifidum* H4	BB_H4	Infant feces
*Bifidobacterium bifidum* H5	BB_H5	Infant feces
*Bifidobacterium bifidum* H22	BB_H22	Infant feces
*Bifidobacterium pseudocatenulatum* YA	BP_YA	Infant feces
*Bifidobacterium longum* S34	BL_S34	Infant feces
*Bifidobacterium longum* subsp. *infantis* ATCC 15697	BI_15697	China Center of Industrial Culture Collection
*Bifidobacterium longum* subsp. *infantis* M63	BI_ M63	Probiotic ^1^
*Escherichia coli* O157: H7 ATCC 35150	EC_O157	Luwei Technology Co. (Shanghai, China)
*Cronobacter sakazakii* ATCC 29544	CS_29544	Luwei Technology Co. (Shanghai, China)
*Salmonella enteritidis* CMCC (B) 50335	SE_50335	Luwei Technology Co. (Shanghai, China)

^1^ *Bifidobacterium longum* subsp. *infantis* M63 (BI_M63) was isolated from commercial ProTectis drops (Bio Gaia, Stockholm, Sweden).

All tested bifidobacteria were anaerobically cultured at 37 °C in de Man, Rogosa and Sharpe (MRS, Qingdao Hope Bio-Technology Co., Ltd., Qingdao, China) broth supplemented with 0.05% (*w*/*v*) L-cysteine (MRSC). Enteric pathogens were sub-cultured twice in Luria–Bertani (LB) broth (Qingdao Hope Bio-Technology Co., Ltd., Qingdao, China) at 37 °C for 24 h. All bifidobacterial strains used in adhesion assays were harvested from overnight cultures (approximately 16–18 h) at the stationary growth phase to ensure consistency in bacterial surface characteristics across experiments.

### 2.2. Surface Hydrophobicity of Tested Bifidobacteria

Surface hydrophobicity of bifidobacteria was assessed as previously described [20]. Bacterial suspensions were prepared by centrifugation at 6000× *g* for 10 min at 4 °C, followed by two washes with phosphate-buffered saline (PBS, pH 7.2, Solarbio Science & Technology Co., Ltd., Beijing, China). The bifidobacterial cells were resuspended in PBS to an optical density (OD_600nm_) of 0.8. Three milliliters of bacterial suspension was mixed with 1 mL of xylene, vortexed for 2 min, and incubated at 37 °C for 120 min without agitation. The OD_600nm_ of the aqueous phase was measured, with PBS serving as the control. Hydrophobicity was determined as follows:

$$Hydrophobicity(\%)=\left[\left(A_{0}-A_{t}\right)/A_{0}\right]\times 100\%$$

where *A*_0_ and *A_t_* represent the values of OD_600nm_ before mixing and after incubation.

### 2.3. Auto-Aggregation Assay of Bifidobacteria

Auto-aggregation capacity was determined as previously described [20]. Bacterial suspensions in PBS were prepared as described above. Four milliliters of the suspension were vortexed for 2 min and incubated at 37 °C for 5 h. The supernatant absorbance at 600 nm was measured before (*A*_0_) and after (*A_t_*) incubation, and auto-aggregation was calculated using the given formula.

$$\mathrm{Auto-aggregation}\left(\%\right)=\left(1-\frac{A_{t}}{A_{0}}\right)\times 100\%$$

### 2.4. Adhesion of Tested Bifidobacteria to Caco-2 Cells

The adhesion of bifidobacteria to Caco-2 cells was evaluated as previously described [21]. Briefly, Caco-2 cells (Procell Life Science & Technology Co., Ltd., Wuhan, China) were cultured in Dulbecco’s modified Eagle’s medium (DMEM) supplemented with 10% fetal bovine serum (FBS), 1% non-essential amino acids and 100 U/mL penicillin–streptomycin in 24-well plates. Cells were maintained at 37 °C with 5% CO_2_, following standard protocols [22].

Overnight bifidobacterial cultures in MRSC broth were centrifuged, washed and adjusted to 10^7^ CFU/mL in complete DMEM. Bacteria were added to Caco-2 cell monolayers in 24-well plates at a multiplicity of infection of 100:1 and incubated for 120 min at 37 °C under 5% CO_2_. After incubation, non-adherent bacteria were removed by washing the wells three times with PBS. Adherent bacteria were released with trypsin (3 min), transferred to sterile tubes, and then centrifuged at 6000× *g* for 10 min at 4 °C. The recovered bacteria were resuspended in 1 mL PBS, serially diluted and plated on MRS agar, followed by anaerobic incubation at 37 °C for 48 h. The adhesion rate was calculated as:

$$Adhesion\;rate\left(\%\right)=V_{t}/V_{0}\times 100\%$$

where *V_0_* and *V_t_* represent the bifidobacterial counts before and after adhesion to the Caco-2 cells, respectively.

### 2.5. Mucin Adhesion Assay

Bifidobacterial adhesion to immobilized mucin was assessed in 96-well microtiter plates [23]. Wells were coated overnight at 4 °C with 300 µL of porcine mucin (0.5 mg/mL, Shanghai Ryon Biological Co., Ltd., Technology, Shanghai, China) in sterile PBS, then washed twice with PBS to remove unbound mucin. Bacterial cultures were harvested by centrifugation (6000× *g* for 5 min, at 4 °C), washed twice with PBS, and adjusted to 10^8^ CFU/mL in PBS. Two hundred microliters of bacterial suspensions were added to their designated wells and incubated at 37 °C for 120 min to allow adhesion. Wells were washed five times with PBS to remove non-adherent cells. Adherent cells were released by adding 100 µL of 0.05% (*v*/*v*) Triton X-100 (Biotopped Science & Technology Co., Ltd., Beijing, China) and incubating for 20 min at 37 °C. The resulting suspension was diluted in PBS, plated on MRSC agar, and enumerated.

### 2.6. Identification of Key Adhesins on Bifidobacterial Surfaces

Overnight cultures of bifidobacteria were washed twice with PBS, and surface layer proteins (SLPs) and lipoteichoic acid (LTA) were removed using Yuan’s method [24]. Bacteria were treated with 5 M LiCl at 37 °C and 200 rpm for 30 min, followed by three PBS washes and centrifugation at 12,000× *g*, 4 °C for 15 min to remove SLPs. To eliminate LTA, cells were incubated in 2% (*w*/*v*) bovine serum albumin at 37 °C, 200 rpm for 30 min, washed three times with PBS, and centrifuged at 4500× *g*, 4 °C for 10 min.

EPS was extracted according to Zhang’s method [25] to assess its impact on bacterial adhesion to mucin. Bacterial suspensions were centrifuged at 8000× *g*, 4 °C for 10 min, and the supernatant was collected. Trichloroacetic acid was added to the supernatant to achieve a concentration of 40 mg/mL, and the mixture was incubated at 4 °C for 8 h. EPS was further precipitated by adding three volumes of 95% ethanol to the supernatant and storing the mixture at 4 °C for 15 h. The precipitate was collected by centrifugation at 10,000× *g*, 4 °C for 10 min and resuspended in 1 mL distilled water. The EPS solution was then combined with the bacterial suspension.

Mucin adhesion was reassessed following SLP or LTA removal or EPS supplementation.

### 2.7. Scanning Electron Microscopy (SEM) Analysis of Bifidobacteria

Bifidobacteria were washed three times with sterile water, prepared following a published protocol [18], and examined via a Hitachi S-3400N scanning electron microscope (SEM) (Hitachi High Technologies, Tokyo, Japan).

### 2.8. Co-Aggregation Assay for Bifidobacteria and Pathogens

The co-aggregation of bifidobacteria and pathogens was assessed following the protocol outlined in a previous study [26]. Briefly, bifidobacteria and pathogenic strains (EC_O157, CS_29544, and SE_50335) were washed twice with PBS, centrifuged at 6000× *g* for 10 min at 4 °C, and resuspended in sterile PBS to an optical density (OD_600nm_) of 0.8. Equal volumes (500 µL each) of bifidobacteria and pathogens were combined and incubated at 37 °C with agitation (100 rpm) for 5 h. The mixtures were Gram-stained and examined microscopically.

### 2.9. Competitive Adhesion Assay of Bifidobacteria and Pathogens

The inhibitory effect of bifidobacteria on pathogen adhesion to mucin was assessed as previously described [27]. Bifidobacteria and pathogens were prepared as above, with cell densities adjusted to 10^8^ CFU/mL in sterile PBS. For the assay, 100 µL each of bifidobacteria and pathogens were simultaneously added to mucin-coated wells and incubated for 120 min. Unbound bacteria were removed by washing each well five times with 200 µL of sterile PBS, and adherent bacteria were detached using 100 µL of 0.05% Triton X-100 (*v*/*v*) for 20 min at 37 °C. Pathogen adhesion was quantified by plating on LB agar, and adhesion competition was calculated as the difference in pathogen adhesion in the absence and presence of bifidobacteria.

### 2.10. Exclusion Inhibition of Pathogen Adhesion to Mucin by Bifidobacteria

The inhibitory effect of bifidobacteria on pathogen adhesion was assessed as previously described [28]. Bifidobacteria and pathogens were prepared as outlined above. A 100 µL aliquot of bifidobacterial suspension was added to mucin-coated 96-well plates and incubated at 37 °C for 60 min. Wells were washed three times with PBS, followed by the addition of a 100 µL pathogen suspension and incubation at 37 °C for 60 min. Non-adherent pathogens were removed through three PBS washes. Adherent bacteria were released by treating with 100 µL of 0.05% Triton X-100 for 20 min, and pathogen counts were determined by plating on LB agar. Adhesion inhibition was quantified as the percentage reduction in pathogen adhesion in the presence of bifidobacteria compared to the absence of bifidobacteria.

### 2.11. Displacement of Pathogen Adhesion to Mucin by Bifidobacteria

The displacement capacity of bifidobacteria against pre-adhered pathogens was assessed as described previously [28], with the modification that pathogens were allowed to adhere before introducing bifidobacteria.

### 2.12. Statistical Analysis

All experiments were performed in three independent biological replicates, and data are expressed as the mean ± standard deviation. Statistical analyses were carried out using Duncan’s multiple range test in SPSS v. 27. Values with different lowercase letters are significantly different (*p* < 0.05).

## 3. Results

### 3.1. Hydrophobicity and Auto-Aggregation Capacity of Bifidobacteria

**Figure 1:**
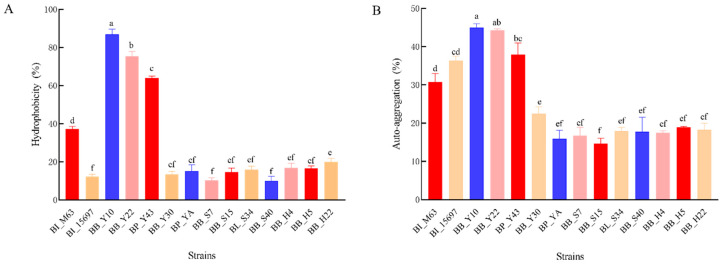
Surface hydrophobicity (**A**) and auto-aggregation (**B**) capacity of the tested bifidobacteria after 5 h of incubation. Data are expressed as mean ± standard deviation (SD), Different lowercase letters indicate significant differences among strains (*p* < 0.05).

The hydrophobicity and auto-aggregation properties of 12 bifidobacterial isolates from infant feces were analyzed, with BI_15697 and BI_M63 serving as controls (Figure 1). Bifidobacteria were categorized into three clusters based on hydrophobicity (Figure 1A). Cluster 1 exhibited the highest hydrophobicity. BB_Y10 (86.84%), BB_Y22 (75.46%), and BP_Y43 (64.30%) showed significantly higher hydrophobicity than controls BI_M63 and BI_15697, as well as other strains (*p* < 0.05). BI_M63 displayed moderate hydrophobicity (37.32%) and was placed in cluster 2, while the remaining strains had minimal hydrophobicity, comparable to BI_15697 in cluster 3 (*p* < 0.05).

Auto-aggregation capacity mirrored hydrophobicity patterns (Figure 1B). BB_Y10 and BB_Y22 exhibited the highest auto-aggregation rates (44.95% and 44.25%, respectively), significantly surpassing the controls BI_M63 and BI_15697 (*p* < 0.05). All other strains (BP_YA, BB_S7, BB_S15, BB_Y30, BL_S34, BB_S40, BB_H4, BB_H5, and BB_H22) demonstrated weak auto-aggregation. These findings suggest a positive correlation between hydrophobicity and auto-aggregation [29].

### 3.2. Adhesion of Bifidobacterium to Caco-2 Cells and Mucin

Based on adhesion to Caco-2 cells, bifidobacteria were categorized into three groups (Table 2). Group I exhibited the highest adhesion, with BB_Y22, BB_Y10, and BB_S15 displaying rates of 19.20 ± 0.72%, 15.70 ± 1.31%, and 15.30 ± 1.49%, respectively. Group II showed moderate adhesion, with BB_Y30 and BB_H5 demonstrating rates of 10.18 ± 0.84% and 9.66 ± 0.17%, which were significantly higher than those of Group III (*p* < 0.05). The latter included BI_M63, BB_S7, BI_15697, BB_H22, BP_Y43, BL_S34, BB_S40, BB_H4, and BP_YA, with rates between 4.39 ± 0.35% and 8.74 ± 0.59%.

Similarly, mucin adhesion was assessed, dividing bifidobacteria into three groups. Group I (BB_Y22 and BB_Y10) showed significantly stronger adhesion compared to other strains (*p* < 0.05). Group II, represented by control strains BI_15697 and BI_M63, exhibited moderate adhesion. Group III had the weakest adhesion, ranging from 17.93 ± 0.45 CFU/mL to 36.26 ± 2.96 CFU/mL.

Pearson correlation analysis revealed a strong linear relationship (r = 0.70) between adhesion rates to Caco-2 cells and mucin. Based on these findings, strains with high (BB_Y22 and BB_Y10), moderate (BI_15697 and BI_M63), and low (BB_S7) adhesion were selected for further study.

### 3.3. Identification of Key Adhesins on Bifidobacteria Surfaces

To identify the primary cell surface adhesins of *Bifidobacterium*, SLPs and LTA were removed, and EPS was added, with mucin adhesion assessed pre- and post-treatment. BI_M63 exhibited a 99.39 ± 0.08% reduction in adhesion capacity upon SLP removal, the largest decline among the five tested strains (*p* < 0.05) (Table 3). BB_Y22 showed a 41.84 ± 6.84% reduction, whereas BI_15697, BB_Y10, and BB_S7 had negligible changes in adhesion. These results suggest that SLPs are critical for BI_M63 and BB_Y22 adhesion but has minimal impact on the other strains, likely due to variations in SLP structure, distribution, and type [30].

LTA removal significantly decreased BI_M63 and BB_Y22 adhesion by 99.77 ± 0.03% and 67.99 ± 0.96%, respectively (Table 4). BB_Y10 exhibited a moderate 18.02 ± 2.65% reduction, while BB_S7 and BI_15697 showed negligible change, likely due to inherently low adhesion. These findings highlight LTA’s significant role in BI_M63, BB_Y22, and BB_Y10 adhesion to mucin.

When EPS was introduced, all strains exhibited improved adhesion to varying degrees (Table 5). BB_Y22, BI_M63, and BB_Y10 showed marked increases of 43.38 ± 2.65%, 35.98 ± 4.34%, and 31.58 ± 2.23%, respectively. BI_15697 had a moderate increase of 24.27 ± 4.63%, while BB_S7 recorded a minor improvement of 2.00 ± 0.44%. These results indicate that EPS may contribute to bacterial adhesion, possibly by acting as a bridging molecule or by modifying surface properties, thereby potentially aiding strain colonization in diverse environments.

### 3.4. Bifidobacterium Morphology Observed via SEM

**Figure 2:**
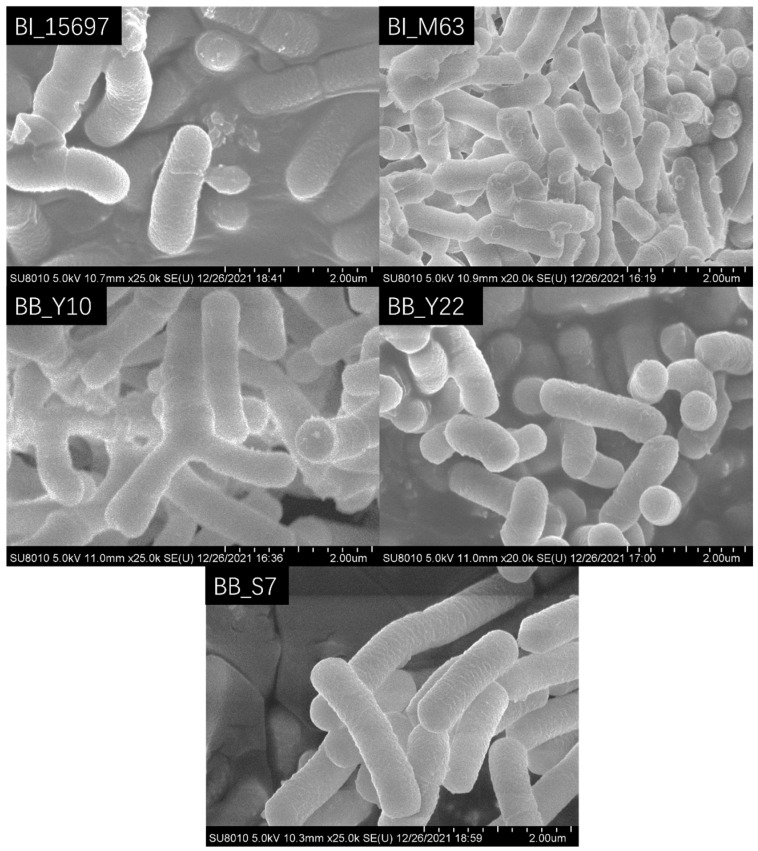
Observation of the morphology of bifidobacterial (BI_M63, BI_15697, BB_Y10, BB_Y22 and BB_S7) by scanning electron microscope (SEM).

The morphology of five bifidobacterial strains observed by SEM is shown in Figure 2. SEM analysis of the five bifidobacteria revealed significant morphological variation, as shown in Figure 2. 

BB_Y10 appeared as a bifid or blunt-ended rod, while BB_Y22 exhibited a curved rod shape with a rounded structure. Both strains displayed irregular morphology, rough surfaces, and aggregation tendencies. While these morphological features are qualitatively consistent with the observed strong auto-aggregation, surface hydrophobicity, and adhesive capabilities of these strains, SEM observations alone cannot establish a causal relationship between surface morphology and adhesive properties. Further quantitative and biochemical analyses would be required to confirm such associations.

### 3.5. Co-Aggregation Assays of Pathogens with Probiotic Strains

**Figure 3:**
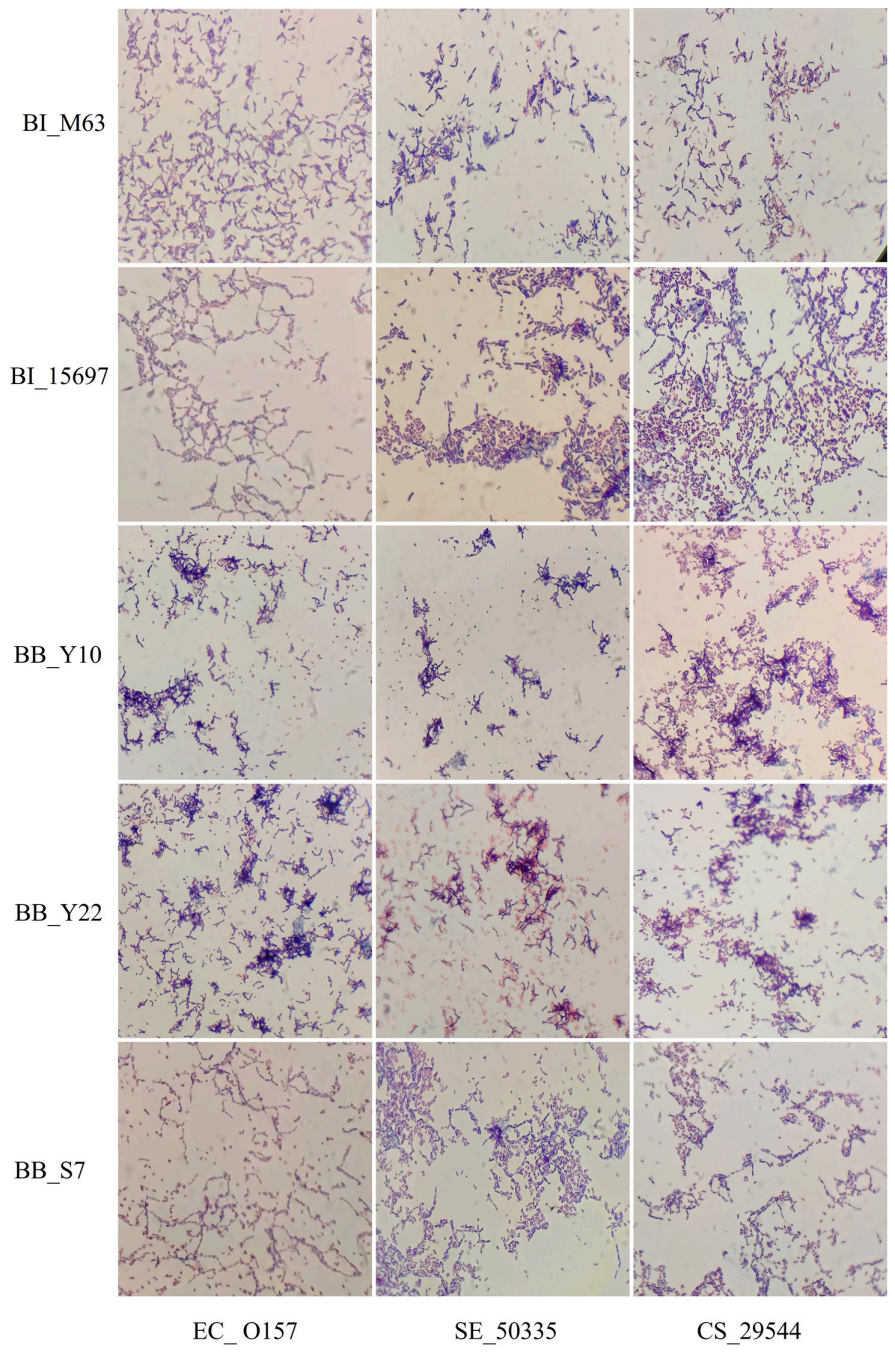
Bifidobacteria (BI_M63, BI_15697, BB_Y10, BB_Y22, and BB_S7) co-aggregated with EC_O157, SE_50335, and CS_29544, respectively, was observed by microscopy.

Figure 3 illustrates the co-aggregation of pathogens (EC_O157, SE_50335, and CS_29544) with selected bifidobacteria. BB_Y10 and BB_Y22 effectively co-aggregated with all three enteric pathogens. In contrast, BI_15697, BI_M63, and BB_S7 showed weaker co-aggregation and remained predominantly dispersed.

### 3.6. Antagonistic Adhesion of Bifidobacteria to Pathogenic Bacteria

**Figure 4:**
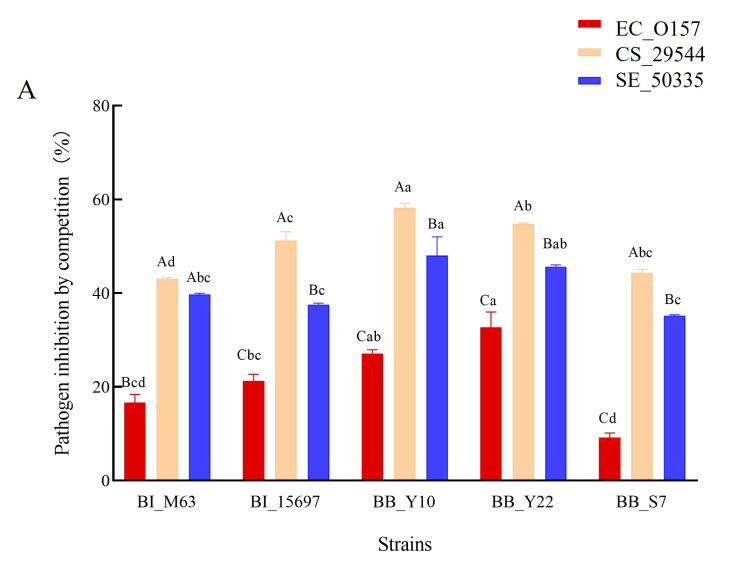

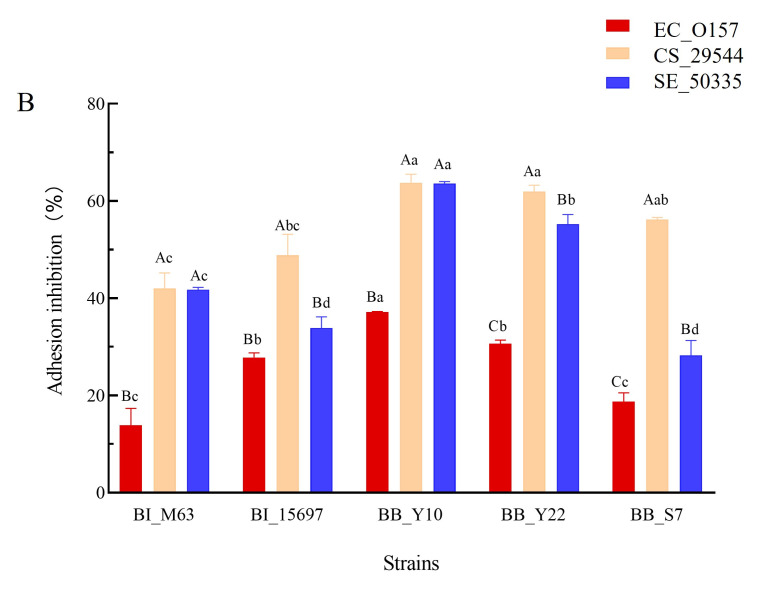

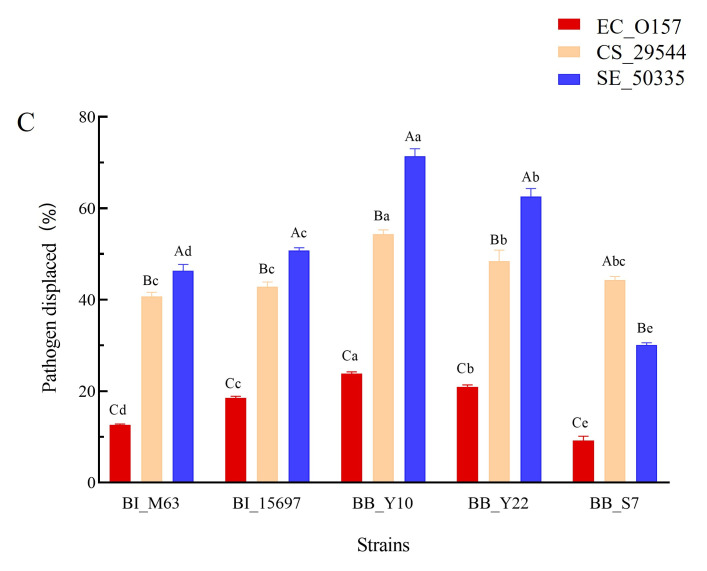
Competitive inhibition of EC_O157, CS_29544 and SE_50335 to mucin by bifidobacteria in a microtiter plate assay (**A**). Inhibition of the adhesion of EC_O157, CS_29544 and SE_50335 to intestinal mucus due to the presence of previously adhered bifidobacteria (**B**). Displacement of adherent EC_O157, CS_29544 and SE_50335 from mucus by bifidobacterial (**C**). Results are expressed as the mean ± standard deviation (mean ± SD). Lowercase letters indicate inhibition of the same pathogen by different bifidobacterial strains, while uppercase letters indicate inhibition of distinct intestinal pathogens by same bifidobacterial strains.

The antagonistic effects of bifidobacteria on pathogen adhesion were evaluated using three methods: competitive, exclusion, and displacement inhibition (Figure 4). The tested bifidobacteria demonstrated greater inhibitory effects against CS_29544 compared to SE_50335 and EC_O157 in competitive inhibition (Figure 4A). BB_Y10 and BB_Y22 exhibited the strongest inhibition, reducing CS_29544 adhesion to mucin by 58.20% and 54.81%, respectively (*p* < 0.05), compared to BI_15697 (43.08%), BI_M63 (51.27%), and BB_S7 (52.31%). Additionally, BB_Y10 and BB_Y22 more effectively inhibited the adhesion of SE_50335 and EC_O157 than the other strains evaluated.

In exclusion assays, all bifidobacteria showed stronger inhibition of CS_29544 adhesion than of SE_50335 or EC_O157 (Figure 4B). BB_Y10 and BB_Y22 displayed the highest exclusion effects, reducing CS_29544 adhesion by 63.75% and 61.96%, respectively (*p* < 0.05), while BI_15697, BI_M63, and BB_S7 showed lower inhibition rates of 42.03%, 48.85%, and 56.17%, respectively. These findings suggest that the exclusion ability of bifidobacteria is closely linked to their adhesion capabilities.

The displacement of pathogens already attached to mucin also depended on the bifidobacterial strain [24]. Unlike competitive inhibition, bifidobacteria were most effective at displacing SE_50335 (Figure 4C). BB_Y10 and BB_Y22 displaced SE_50335 by 71.41% and 62.57%, respectively (*p* < 0.05), significantly outperforming BI_15697, BI_M63, and BB_S7. The strains displayed moderate displacement efficacy against CS_29544, with BB_Y10 and BB_Y22 reducing adhesion by 54.35% and 48.45%, respectively (*p* < 0.05), while BB_S7, BI_15697, and BI_M63 showed weaker effects. All strains showed the least ability to displace EC_O157.

## 4. Discussion

Adhesion to and colonization of intestinal epithelial cells are key criteria for probiotic selection, as highly adherent bifidobacteria can competitively exclude intestinal pathogens [31]. Initial adhesion may involve nonspecific physical forces (e.g., hydrophobic interactions), followed by specific interactions with cell wall components [26].

Cellular hydrophobicity is essential for probiotics to exclude intestinal pathogens, adhere to epithelial cells, and colonize the gastrointestinal tract [32]. Auto-aggregation plays a key role in enterocyte adhesion and blocking pathogen colonization [33]. Strains with higher auto-aggregation and surface hydrophobicity exhibit greater adhesion capacity [34]. Among the strains tested, BB_Y10 and BB_Y22 displayed superior hydrophobicity and auto-aggregation, indicating potential for specific binding in the gastrointestinal tract.

Probiotic adhesive properties are commonly assessed using human colorectal adenocarcinoma cell lines like Caco-2, HT-29, and mucin immobilized on abiotic surfaces [33]. The Caco-2 cell model is particularly advantageous because these cells undergo morphological and functional differentiation in vitro, mimicking mature intestinal epithelial cells. This makes the model ideal for studying bacterial and viral adhesion to enterocytes [35]. Intestinal epithelial cells are shielded by a mucus layer composed of glycoproteins called mucin, which serve as the initial site of bacterial adhesion [36]. Since bacteria interact with mucin before accessing the intestinal epithelium, immobilized mucin adhesion models provide an efficient screening platform for probiotic strains, overcoming the limitations of complex cell culture systems.

In this study, results from the Caco-2 cell adhesion assay closely aligned with those from the mucin adhesion assay (Table 2). The comprehensive comparative data presented in Table 2 are particularly valuable, as they demonstrate consistent strain ranking across both adhesion models: BB_Y22 and BB_Y10 exhibited the highest adhesion to both Caco-2 cells (19.20% and 15.70%, respectively) and mucin (103.06 × 10^5^ and 73.87 × 10^5^ CFU/mL, respectively), while BB_S7 showed the weakest adhesion in both systems (5.90% and 27.89 × 10^5^ CFU/mL, respectively). The strong correlation between the two models (r = 0.70) supports the suitability of mucin as a practical preliminary screening system for probiotic adhesion. Given that mucin serves as the initial adhesion site for bifidobacteria in the colonizing gut and is cost-effective and simple to prepare [36], and considering that Caco-2 cell assays are technically demanding, time-consuming, and require specialized facilities, mucin-based screening offers a robust and accessible alternative for initial strain evaluation. Therefore, the agreement observed in Table 2 across these two distinct but complementary models provides compelling evidence that mucin adhesion assays can reliably predict Caco-2 cell adhesion performance, enabling efficient prioritization of candidate strains for subsequent in-depth characterization. Based on these findings, strains with high (BB_Y22 and BB_Y10), moderate (BI_15697 and BI_M63), and low (BB_S7) adhesion were selected for subsequent experiments to comprehensively analyze factors influencing *Bifidobacterium* adhesion and its antagonistic effects on intestinal pathogen adhesion.

Bifidobacteria enter the GI tract and interact with the host via bacterial surface molecules called adhesins, which are strain- or species-specific [4]. Surface layer proteins (SLPs), lipoteichoic acid (LTA), and extracellular polysaccharides (EPS) are important adhesins.

SLPs are proteins located in the outermost layer of the bacterial cell wall that facilitate adhesion to the intestinal mucosa. As structural components, SLPs serve various functions, including resisting pathogen virulence, acting as antifouling coatings, and promoting adhesion [37]. LTA assists bifidobacteria in binding to intestinal epithelial cells [38]. Bifidobacteria contain substantial LTA in their cell walls and continuously release it into the extracellular environment during growth [39]. Bifidobacterial EPS are large carbohydrate polymers secreted into the extracellular environment, composed of monosaccharides linked by glycosidic bonds, and exhibit complex structures with diverse functions. EPS facilitate communication between colonized probiotics and host cells, enhancing probiotic survival in the gastrointestinal tract through adhesion to the mucosal epithelium [40].

Changes in bifidobacterial adhesion to mucin following SLP or LTA removal, or EPS supplementation, demonstrate that adhesion mechanisms are highly strain-specific. SLPs significantly influence mucin adhesion in BI_M63 and BB_Y22, with reductions of 99.39% and 41.84%, respectively, upon removal, unlike in BI_15697, BB_Y10, and BB_S7. LTA is essential for adhesion in BI_M63 and BB_Y22, decreasing adhesion by 99.77% and 67.98%, respectively, but has a moderate effect on BB_Y10 (17.90% reduction) and a negligible impact on BB_S7 and BI_15697. EPS supplementation markedly increased adhesion across all strains, with the highest gains (31–43%) observed in BB_Y22, BI_M63, and BB_Y10. In conclusion, adhesion in BI_M63 appears to be primarily regulated by SLPs and LTA, suggesting coordinated surface interactions. BB_Y22 adhesion may be mediated by all three factors, whereas BB_Y10 adhesion appears to be more dependent on EPS, with minimal SLP contribution. While EPS supplementation enhanced adhesion in most strains, further studies are needed to determine whether EPS acts directly as an adhesin or indirectly by modifying surface properties and to elucidate the underlying molecular mechanisms.

It is important to interpret the in vitro adhesive performance of BI_M63 in the context of its documented in vivo efficacy. BI_M63 is a well-characterized commercial probiotic strain with established beneficial effects in clinical settings, including alleviation of abdominal pain and improvement in quality of life in children with irritable bowel syndrome [41], reduction in allergic symptoms in pediatric populations [42], and modulation of gut microbiota composition in healthy infants [43]. The discrepancy between the robust in vivo efficacy demonstrated in existing studies and the moderate in vitro adhesion observed in our study may reflect the multifactorial nature of probiotic colonization in vivo, which involves not only adhesion but also competitive exclusion, immune modulation, and metabolic interactions with the host and indigenous microbiota [38]. Therefore, the pronounced dependence of BI_M63 on SLPs and LTA for in vitro adhesion should be interpreted as identifying key functional molecules that may contribute to its colonization capacity, rather than as a limitation of its probiotic potential. The distinct surface molecular requirements of BI_M63, compared with those of the HMO-metabolizing isolates BB_Y10 and BB_Y22, underscore the strain-specific diversity of adhesion mechanisms among bifidobacteria and highlight that different strains may achieve successful gut colonization through distinct molecular strategies.

SEM revealed that BI_15697 and BI_M63 exhibit relatively regular rod-shaped structures with minimal visible surface material, while strains BB_Y22 and BB_Y10, which demonstrated higher adhesion abilities in quantitative assays, appeared to display more abundant surface-associated material. We speculate that these surface-associated materials may represent extracellular substances; however, definitive identification of their nature as specific extracellular components (e.g., EPS, SLPs, or LTA) requires further biochemical or immunological characterization. Adhesion assays revealed that components of the extracellular matrix, such as SLPs, LTA, and EPS, are associated with enhanced adhesion and may promote intestinal colonization, potentially improving survival on the gastrointestinal surface [44,45] and contributing to the formation of an immune barrier against pathogenic infections [46].

Probiotics with high adhesion capacity can co-aggregate with pathogens, preventing their intestinal attachment [29,47]. The inhibition of pathogen adhesion by bifidobacteria is a noteworthy application of their protective effects. Competition between probiotics and pathogens arises when resources such as niches and nutrients are limited [48]. It has been reported that certain bifidobacteria are able to inhibit the mucosal adhesion of *E. coli* and *Salmonella* [49]. This study demonstrates that BB_Y22 and BB_Y10 exhibit competitive adhesion, notable co-aggregation, and robust antagonistic effects against pathogenic bacteria. However, BB_S7 failed to effectively inhibit pathogen adhesion, indicating that adhesion capacity is strain-specific [50].

Adhesion is a population-dependent phenomenon, necessitating sufficient strain density to achieve optimal adhesion [51]. Adherent bifidobacteria reduce the risk of pathogen colonization via a priority colonization mechanism, while a high bifidobacterial density forms a “biological barrier” that impedes pathogen access to intestinal epithelial cells [52]. In this study, BB_Y22 and BB_Y10 demonstrated the capacity to exclude pathogens in the mucin adhesion model, suggesting a potential mechanism by which these strains may form biofilms and establish a mucus barrier in vivo to inhibit pathogen adhesion. This hypothesis requires validation through in vivo studies, as the in vitro Caco-2 and mucin models cannot fully replicate the dynamic and complex environment of the human gastrointestinal tract [53,54].

Bifidobacteria displace pre-adhered pathogens by strengthening the intestinal barrier and boosting host immunity through the stimulation of epithelial secretion of antimicrobial peptides (e.g., defensins and lysozyme) and mucin proteins (MUC2), facilitating the growth of symbiotic microbiota [55,56]. They further suppress pathogen growth and adhesion by iron sequestration in the gut [57]. Current and previous studies have also shown that bifidobacteria can displace pre-colonized pathogens in vitro [58]. While our in vitro data indicate that strains BB_Y10 and BB_Y22 can displace pre-adhered pathogens, such antagonistic activity remains to be validated in vivo. It should nevertheless be noted that these in vitro models fail to fully recapitulate the complex, dynamic environment of the human gastrointestinal tract. Factors such as peristalsis, gastric acid, bile salts, immune responses, and competitive microbiota interactions, all of which may modulate bacterial adhesion in vivo, were not considered in the present study. Therefore, the probiotic potential of BB_Y10 and BB_Y22 warrants further validation through in vivo models, such as gnotobiotic animal studies or human intervention trials, to confirm their colonization efficacy and pathogen exclusion capacity under physiological conditions.

## 5. Conclusions

In summary, bifidobacterial adhesion is a multifaceted process influenced by auto-aggregation, surface hydrophobicity, and surface-associated molecules. Using in vitro Caco-2 cell and mucin models, we found consistent adhesion results across assays, though these models cannot fully replicate the dynamic gastrointestinal environment. Distinct morphologies and surface roughness among strains may contribute to strain-specific adhesion differences. Surface-associated molecules, including surface layer proteins, extracellular polysaccharides, and lipoteichoic acid, appear to differentially influence adhesion in vitro, but their specific mechanisms require further investigation. Pathogen antagonism was strain-specific, with *B. bifidum* Y10 and Y22 showing the strongest in vitro adhesion and pathogen inhibition. These findings suggest probiotic potential; however, the in vivo colonization capacity and pathogen displacement efficacy of these strains remain to be validated. Future studies should include animal or clinical trials to confirm their functional benefits before probiotic development. Furthermore, the strong agreement between the Caco-2 cell and mucin adhesion data supports the utility of mucin-based assays as practical preliminary screening tools for probiotic adhesion, offering a cost-effective alternative to cell culture models.

## Figures and Tables

**Table 2:** Adhesion of bifidobacteria to Caco-2 cells and mucin. Results are expressed as the mean ± standard deviation (mean ± SD). Different lowercase letters show significant differences between different strains in the same column.

Strains	Adhesion to Caco-2 Cells (%)	Adhesion to Mucin (10^5^ CFU/mL)
BI_M63	4.39 ± 0.35 ^g^	42.17 ± 3.97 ^d^
BI_15697	6.29 ± 0.27 ^f^	54.44 ± 3.10 ^c^
BB_Y10	15.70 ± 1.31 ^b^	73.87 ± 7.01 ^b^
BB_Y22	19.20 ± 0.72 ^a^	103.06 ± 4.03 ^a^
BB_Y30	10.18 ± 0.84 ^c^	32.58 ± 8.20 ^ef^
BP_Y43	6.78 ± 0.54 ^ef^	32.94 ± 8.20 ^ef^
BP_YA	8.74 ± 0.59 ^cd^	27.87 ± 3.34 ^fg^
BB_S7	5.90 ± 0.65 ^fg^	27.89 ± 4.48 ^fg^
BB_S15	15.30 ± 1.49 ^b^	36.26 ± 2.96 ^de^
BL_S34	7.01 ± 0.87 ^def^	29.08 ± 0.45 ^efg^
BB_S40	7.73 ± 0.75 ^def^	21.87 ± 1.57 ^gh^
BB_H4	8.46 ± 1.47 ^cde^	17.93 ± 0.45 ^h^
BB_H5	9.66 ± 0.17 ^c^	21.76 ± 2.09 ^gh^
BB_H22	6.36 ± 1.03 ^f^	21.89 ± 2.71 ^gh^

**Table 3:** Changes in the ability of bifidobacterial strains to adhere to mucin before and after removal of surface layer proteins (SLPs) from the bacterium. Results are shown as the mean ± standard deviation. Different lowercase letters show significant differences between different strains in the same column (*p* < 0.05).

Strains	Adhesion Number Before Treatment (10^5^ CFU/mL)	Adhesion Number After Treatment (10^5^ CFU/mL)	Relative Change in Adhesion (%)
BI_M63	43.75 ± 0.71 ^d^	0.27 ± 0.03 ^d^	−99.39 ± 0.08 ^c^
BI_15697	61.66 ± 3.51 ^c^	58.78 ± 4.11 ^ab^	−4.50 ± 3.28 ^a^
BB_Y10	73.38 ± 3.82 ^b^	65.00 ± 5.34 ^a^	−11.32 ± 7.85 ^a^
BB_Y22	94.91 ± 6.05 ^a^	54.88 ± 6.76 ^b^	−41.84 ± 6.84 ^b^
BB_S7	27.81 ± 2.75 ^e^	24.03 ± 1.35 ^c^	−13.09 ± 3.75 ^a^

**Table 4:** Changes in the ability of bifidobacterial strains to adhere to mucin before and after removal of lipoteichoic acid (LTA). Results are shown as the mean ± standard deviation. Different lowercase letters show significant differences between different strains in the same column (*p* < 0.05).

Strains	Adhesion Number Before Removal of LTA (10^5^ CFU/mL)	Adhesion Number After Treatment (10^5^ CFU/mL)	Relative Change in Adhesion (%)
BI_M63	47.76 ± 2.38 ^d^	0.11 ± 0.01 ^c^	−99.77 ± 0.03 ^d^
BI_15697	63.56 ± 4.87 ^c^	60.72 ± 2.98 ^a^	−4.47 ± 8.69 ^b^
BB_Y10	68.38 ± 3.25 ^b^	56.06 ± 5.58 ^a^	−18.02 ± 2.65 ^b^
BB_Y22	88.00 ± 1.41 ^a^	28.17 ± 0.71 ^b^	−67.99 ± 0.96 ^c^
BB_S7	27.89 ± 2.20 ^e^	26.42 ± 2.43 ^b^	−5.27 ± 11.48 ^a^

**Table 5:** Changes in the ability of bifidobacterial strains to adhere to mucin before and after the addition of exopolysaccharides (EPS). Results are shown as the mean ± standard deviation. Different lowercase letters show significant differences between different strains in the same column (*p* < 0.05).

Strains	Adhesion Number Before EPS Supplementation (10^5^ CFU/mL)	Adhesion Number After Treatment (10^5^ CFU/mL)	Relative Increase in Adhesion (%)
BI_M63	35.39 ± 2.64 ^d^	48.17 ± 4.58 ^d^	35.98 ± 4.34 ^b^
BI_15697	51.08 ± 2.95 ^c^	63.41 ± 1.29 ^c^	24.27 ± 4.63 ^c^
BB_Y10	62.28 ± 2.66 ^b^	81.94 ± 3.74 ^b^	31.58 ± 2.23 ^b^
BB_Y22	80.11 ± 1.98 ^a^	114.89 ± 4.50 ^a^	43.38 ± 2.65 ^a^
BB_S7	19.92 ± 1.52 ^e^	20.33 ± 1.64 ^e^	2.00 ± 0.44 ^d^

## Data Availability

The original contributions presented in this study are included in the article. Further inquiries can be directed to the corresponding authors.
